# Association between anthropometric indices and body fat for identifying excess body fat in elementary school children: a population-based cross-sectional study

**DOI:** 10.1186/s40101-025-00410-w

**Published:** 2025-11-19

**Authors:** Kumiko Ohara, Katsuyasu Kouda, Katsumasa Momoi, Tomoki Mase, Yuki Fujita, Akihiro Takada, Yoshimitsu Okita, Harunobu Nakamura

**Affiliations:** 1 Department of Epidemiology for Community Health and Medicine, Kyoto Prefectural University of Medicine, 465 Kajii-Cho, Kawaramachi-Hirokoji, Kamigyo, Kyoto, Kyoto 602-8566 Japan; 2 Department of Hygiene and Public Health, Kansai Medical University, 2-5-1 Shin-Machi, Hirakata, Osaka 573-1010 Japan; 3 Faculty of Psychology and Collaboration, Kyoto Women’s University, 35 Kitahiyoshi-Cho, Imakumano, Higashiyama, Kyoto, Kyoto 605-8501 Japan; 4 Faculty of Human Development and Education, Kyoto Women’s University, 35 Kitahiyoshi-Cho, Imakumano, Higashiyama, Kyoto, Kyoto 605-8501 Japan; 5 Faculty of Health Sciences, Aino University, 4-5-4 Higashi-Ohda, Ibaraki, Osaka 567-0012 Japan; 6 Graduate School of Medical Photonics, Shizuoka University, 3-5-1 Johoku, Chuo-Ku, Hamamatsu, Shizuoka 432-8011 Japan

**Keywords:** Adiposity, Children, Epidemiology, Screening

## Abstract

**Background:**

Identifying and managing obesity in children is essential to prevent obesity-related diseases in adulthood. This study aimed to evaluate the association between body mass index (BMI), degree of obesity, waist circumference, waist-to-height ratio, and body fat—particularly excess body fat.

**Methods:**

Participants included 660 children aged 9–12 years (349 boys and 311 girls). Fat mass, fat-free mass, and body fat percentage were assessed using bioelectrical impedance analysis. The discriminatory ability of BMI, degree of obesity, waist circumference, and waist-to-height ratio to identify excess body fat—defined as body fat percentage exceeding the 85th, 90th, or 95th percentile—was evaluated using receiver operating characteristic (ROC) curve and precision-recall (PR) curve analyses. Classification performance was further evaluated using a confusion matrix, accuracy, precision, recall, F1 score, Cohen’s kappa coefficient, and Matthews correlation coefficient (MCC).

**Results:**

The areas under the ROC curve (AUCs) and 95% confidence intervals (CIs) for BMI, degree of obesity, waist circumference, and waist-to-height ratio in identifying obesity based on body fat percentage were > 0.9 in both sexes in most cases. PR AUCs and 95% CIs for BMI and degree of obesity were ≥ 0.8 in most cases. Precision, recall, and F1 scores for BMI and degree of obesity in identifying obesity at the 85th or 95th percentiles were > 70% in nearly all cases. Kappa coefficients indicated substantial agreement between BMI and the 85th or 90th percentiles of body fat percentage, and moderate agreement for the degree of obesity. The MCC index showed a pattern similar to that of the kappa coefficients.

**Conclusions:**

These findings suggest that BMI and degree of obesity are strongly associated with body fat percentage across the 85th, 90th, and 95th percentiles and that obesity classifications based on BMI as well as degree of obesity align closely with those based on body fat percentage.

**Supplementary Information:**

The online version contains supplementary material available at 10.1186/s40101-025-00410-w.

## Background

Obesity, defined as excessive fat accumulation in the body, poses significant health risks, including diabetes, cardiovascular disease, hypertension, and hyperlipidemia [1–3]. Childhood obesity often persists into adulthood [4, 5]. As childhood obesity continues to rise in low- and middle-income countries and remains prevalent in high-income countries—despite a slowed increase—[6, 7], there is a pressing need to identify and manage obesity in children on a daily basis to help prevent obesity and related diseases in adulthood.

Obesity is commonly assessed using body mass index (BMI), calculated by dividing weight by height squared, and is used as a daily management indicator. In adults, BMI ≥ 25 kg/m^2^ indicates overweight, and BMI ≥ 30 kg/m^2^ indicates obesity [8]. In children, BMI thresholds corresponding to adult values—established by Cole for each sex and age group—are used to define obesity [9]. However, because obesity involves excessive body fat accumulation, assessing body fat directly is more desirable. As BMI does not account for body composition, it may misclassify muscular individuals as obese or individuals with high body fat as being of normal weight [10–13]. Therefore, understanding the relationship between BMI and body fat may improve the interpretation of BMI.

Body fat can be assessed using underwater weighing or dual-energy X-ray absorptiometry (DXA), both of which yield highly correlated measurements [14]. There are some studies that have measured body fat using the DXA method in healthy Japanese children. Kouda et al. used the DXA method to measure body fat in children from fourth to sixth grade students [15]. Additionally, Ohara et al. measured body fat using both the DXA method and the single-frequency BIA method in children from fifth to eighth grade and sixth grade and compared the results of both methods [16]. However, these methods require expensive equipment, making them unsuitable for large-scale, routine use in school settings [17].

Bioelectrical impedance analysis (BIA) offers an alternative approach. While single-frequency BIA has lower accuracy compared with DXA [18–21], recent advances in multi-frequency BIA have improved its accuracy. Several studies have shown that multi-frequency BIA more closely aligns with DXA results [22, 23]. Furthermore, the absence of radiation exposure and increased ease of use make it suitable for generating reference curves of body fat in children and for use in school settings [24–28]. In Japan, there are studies targeting a small number of children’s patients [29, 30]. However, school-based studies are currently almost nonexistent, with the exception of Ohara et al., who reported the results of body fat measurements using the single-frequency BIA method [16].

BMI is commonly used as a simple indicator of obesity in school settings. In Japan, BMI and the degree of obesity—both calculated from height and weight—are used as indicators of obesity in schools. BMI is computed as weight divided by height squared, whereas the degree of obesity is calculated by comparing actual weight with standard weight adjusted for sex, age, and height [31]. Other easily measurable indicators are waist circumference and waist-to-height ratio [32, 33]. However, the extent to which these anthropometric indices reflect body fat remains unclear. Accordingly, this study aimed to clarify the relationship between body fat and simple anthropometric indices—BMI, degree of obesity, waist circumference, and waist-to-hip ratio—especially in cases of excess body fat. Additionally, the present study also examined the performance of these four anthropometric indices in identifying obesity. To achieve this, BMI, degree of obesity, waist circumference, and waist-to-height ratio were compared with BIA-measured body fat using receiver operating characteristic (ROC) curve and precision-recall (PR) curve analyses.

## Methods

### Study design and participants

This cross-sectional survey was conducted from September to November in both 2021 and 2022 in Himeji City, Japan. The source population comprised all 849 children in fourth to sixth grades (441 boys and 408 girls) enrolled at Itohiki and Shirahama Elementary Schools. Among them, 660 students participated in the body composition survey. The discriminatory performance of BMI, degree of obesity, waist circumference, and waist-to-height ratio for identifying excess body fat—defined as body fat percentage exceeding reference values (i.e., 85th, 90th, and 95th percentiles)—was evaluated using ROC and PR curve analyses. This study was conducted in accordance with the Declaration of Helsinki and approved by the ethics committees of Kansai Medical University (ID: 2021250).

### Anthropometric measurements

Body weight was measured to the nearest 0.1 kg using an electronic scale (AD-6351, A & D Company Ltd., Tokyo, Japan), with participants wearing minimal clothing and no shoes. Height was measured to the nearest 0.1 cm using an electronic scale (AD-6351), with participants freestanding without shoes. Methods for measuring waist circumference were previously detailed [34]. In brief, horizontal waist circumference at the umbilicus level was measured at the end of a normal expiration. BMI (kg/m^2^) was calculated as body weight (kg) divided by height squared (m^2^). Degree of obesity (%) was calculated using the formula: (measured weight (kg)—standard weight (kg))/standard weight (kg) * 100 [31]. Waist-to-height ratio was obtained by dividing waist circumference (cm) by height (cm).

### Body fat measurements

Fat mass and body fat percentage were measured using BIA. Measurements were obtained with a multi-frequency BIA system (MC-980-A-N plus, Tanita Corp., Tokyo, Japan) at 1, 5, 50, 250, 500, and 1000 kHz. According to the manufacturer’s instructions, participants stood barefoot on the electrode plate and gripped the handles, ensuring all fingers contacted the electrodes. Arms were kept at the sides without touching the body, and participants were instructed not to move or bend their knees or elbows during measurement. Fat mass, fat-free mass, body fat percentage, and body weight were recorded per the manufacturer’s standard operating procedures. Excess body fat was defined as a body fat percentage exceeding the 85th, 90th, or 95th percentile according to the previous study [35]. Measurements by BIA were performed twice for each participant, and the average value was used. Coefficients of variation between the two measurements were 0.029 for fat mass, 0.007 for fat-free mass, 0.026 for body fat percentage, and 0.002 for body weight.

### Statistical analysis

Two-way analysis of variance (ANOVA) was conducted to examine the effects of sex and grade, as well as their interaction, on body height, body weight, BMI, degree of obesity, waist circumference, waist-to-height ratio, body fat percentage, fat mass, and fat-free mass. The Bonferroni test was used for post hoc analysis. Pearson’s correlation coefficients were calculated for BMI, degree of obesity, waist circumference, waist-to-height ratio, body fat percentage, fat mass, and fat-free mass. For excess body fat determined by body fat percentage, the ability of BMI, degree of obesity, waist circumference, and waist-to-height ratio to discriminate excess fat mass was assessed using ROC and PR curve analyses [36]. Overall accuracy was evaluated using areas under the curve (AUCs) of ROC and PR curves, along with 95% confidence intervals (CIs) [36]. Confusion matrix, accuracy, precision, recall, F1 score, Cohen’s kappa coefficient, and Matthews correlation coefficient (MCC) matrix were also used to evaluate classification performance between obesity determined by BMI, degree of obesity, waist circumference, waist-to-height ratio and obesity determined by body composition. Cohen’s kappa values were classified as follows: 0, poor; 0.00–0.20, weak; 0.21–0.40, fair; 0.41–0.60, moderate; 0.61–0.80, substantial; and 0.81–1.00, almost perfect agreement [37]. MCC values range from − 1 to + 1, with + 1 indicating perfect prediction, 0 indicating random prediction, and − 1 indicating inverse prediction.

All statistical analyses were conducted using SPSS Statistics Desktop for Japan, Version 29 (IBM Japan, Ltd., Tokyo, Japan), and Python Version 3.12.1 (The Python Software Foundation), with *p* < 0.05 considered statistically significant.

## Results

Participant characteristics are summarized in Table 1. The two-way ANOVA showed that both sexes and grades had a significant main effect on weight, BMI, waist circumference, waist-to-height ratio, body fat percentage, fat mass, and fat-free mass. Significant interaction effects between sex and grade were observed for weight, BMI, body fat percentage, and fat mass.

**Table 1:** Participant characteristics

	4th grade		5th grade		6th grade	
	boys (*n* = 124), girls (*n* = 87)		boys (*n* = 111), girls (*n* = 108)		boys (*n* = 114), girls (*n* = 116)	
	Mean	SD	Mean	SD	Mean	SD
Boys						
Height (cm)	137.0	6.2^§^	141.9	7.2^‖^	148.8	7.9^‖, ⁋^
Weight (kg)	33.9	7.6^*,§,‡^	35.3	7.6	41.5	10.9^‖, ⁋^
BMI (kg/m^2^)	17.9	2.9^*,§,‡^	17.4	2.6	18.5	3.7^⁋^
Degree of obesity	3.2	14.6	−2.2	13.3^‖^	0.7	18.6
Waist circumference (cm)	64.2	8.0^†,§,‡^	63.4	8.0	65.4	10.7
WHtR	0.47	0.05^†,§,‡^	0.45	0.05^‖^	0.44	0.06^‖^
Body fat percentage (%)	18.1	8.7^*,†,§^	15.7	8.6^‡^	17.4	11.1^‡^
Fat mass (kg)	6.7	4.6^*,†,§^	6.1	4.6^‡^	8.2	8.6^‡, ⁋^
Fat-free mass (kg)	27.3	3.5^†,§^	29.2	3.9^‖^	33.2	4.8^‡, ‖, ⁋^
Girls						
Height (cm)	136.1	6.9	143.6	6.7^‖^	150.3	5.9^‖, ⁋^
Weight (kg)	31.4	6.6	36.4	7.5^‖^	42.6	7.1^‖, ⁋^
BMI (kg/m^2^)	16.8	2.3	17.5	2.5	18.8	2.4^‖, ⁋^
Degree of obesity	− 0.7	11.8	− 1.1	13.0	0.7	12.4
Waist circumference (cm)	60.7	6.7	62.9	7.1	64.8	6.2^‖^
WHtR	0.45	0.04	0.44	0.04	0.43	0.04
Body fat percentage (%)	18.5	6.1	20.0	6.6	23.7	6.2^‖, ⁋^
Fat mass (kg)	6.1	3.4	7.7	4.2	10.4	4.4^‖, ⁋^
Fat-free mass (kg)	25.2	3.7	28.6	3.7^‖^	32.0	3.2^‖, ⁋^

*BMI* body mass index, *WHtR *waist-to-height ratio

^*^Significant interaction effect between sex and grade (*p* < 0.05, Two-way analysis of variance)

^†^Significant main effect of sex (*p* < 0.05, Two-way analysis of variance)

^§^Significant main effect of grade (*p* < 0.05, Two-way analysis of variance)

^‡^Significantly different from girls in the corresponding grade (*p* < 0.05, Bonferroni test for post-hoc test)

^‖^Significantly different from 4th grade (*p* < 0.05, Bonferroni test for post-hoc test)

^⁋^Significantly different from 5th grade (*p* < 0.05, Bonferroni test for post-hoc test)

Table 2 presents Pearson’s correlation coefficients. Most correlation coefficients between BMI, degree of obesity, waist circumference, and waist-to-height ratio and both body fat percentage and fat mass were > 0.8 in both sexes, except for waist-to-height in girls. In contrast, the correlation coefficients between fat-free mass and these anthropometric indices were significant but lower than those for body fat percentage and fat mass (range of correlation coefficients on fat-free mass: 0.243–0.727).

**Table 2:** Pearson’s correlation coefficients between body composition and anthropometric status

	Boys (*n *= 349)				Girls (*n *= 311)			
	BMI (kg/m^2^)	DO	WC (cm)	WHtR	BMI (kg/m^2^)	DO	WC (cm)	WHtR
Body fat percentage (%)	.958^**^	.936^**^	.937^**^	.913^**^	.945^**^	.844^**^	.888^**^	.713^**^
Fat mass (kg)	.954^**^	.887^**^	.937^**^	.839^**^	.941^**^	.777^**^	.888^**^	.627^**^
Fat-free mass (kg)	.504^**^	.243^**^	.507^**^	.113^*^	.727^**^	.399^**^	.670^**^	.187^**^

*BMI* body mass index, *DO* degree of obesity, *WC* waist circumference, *WHtR* waist-to-height ratio

*p < 0.05

^**^p < 0.01 (Pearson’s correlation coefficient)

**Fig. 1:**
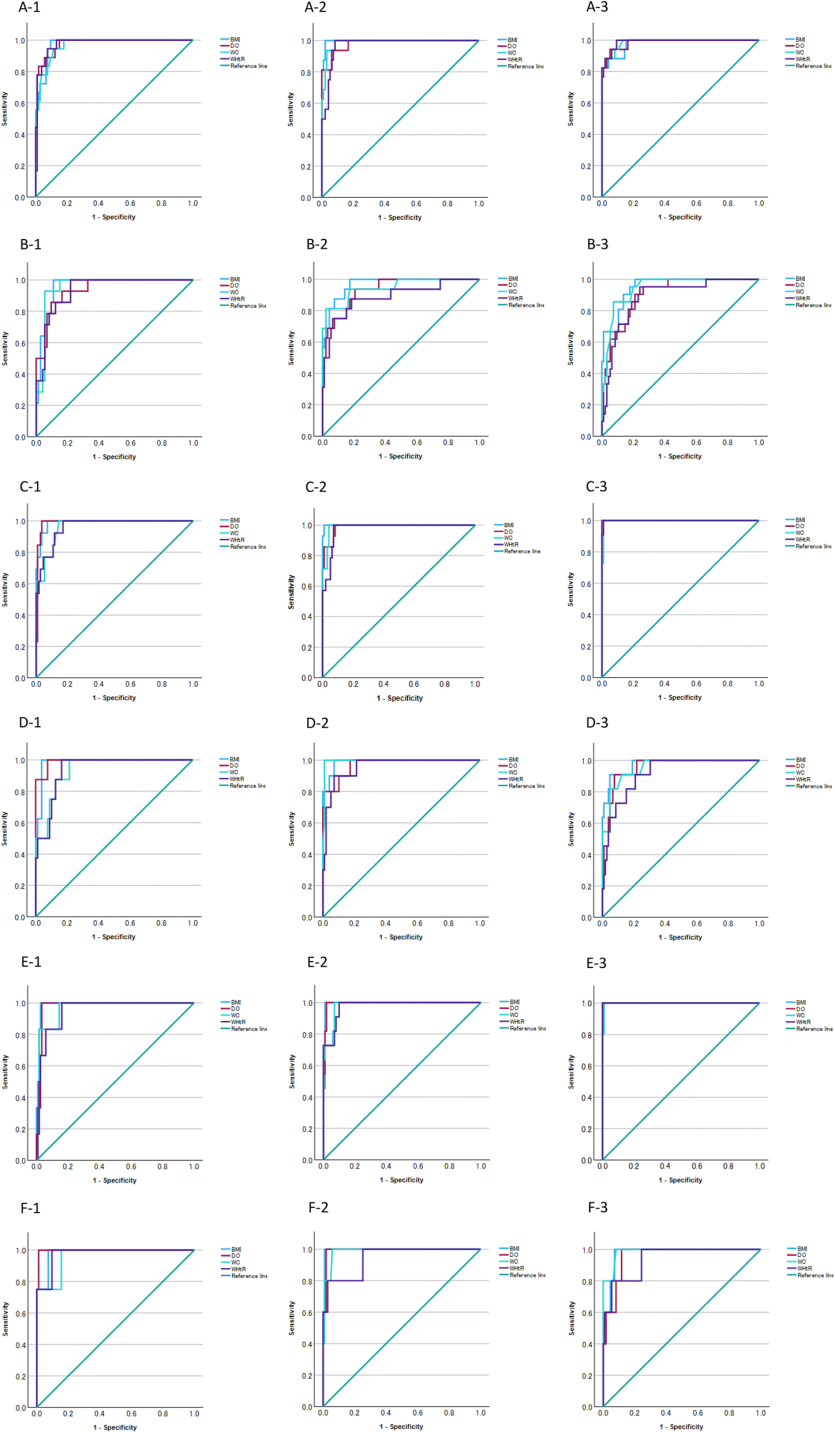
Receiver operating characteristic curves for body mass index (blue line), degree of obesity (red line), waist circumference (light blue line), and waist-to-height ratio (purple line) to identify obesity as determined by body fat percentage. A-1 to A-3 and B-1 to B-3, 85th percentile of body fat percentage; C-1 to C-3 and D-1 to D-3, 90th percentile of body fat percentage; and E-1 to E-3 and F-1 to F-3, 95th percentile of body fat percentage. Grade and sex are as follows: A-, C-, and E-1, 4th grade boys; A-, C-, and E-2, 5th grade boys; A-, C-, and E-3, 6th grade boys; B-, D-, and F-1, 4th grade girls; B-, D-, and F-2, 5th grade girls; and B-, D-, and F-3, 6th grade girls

**Fig. 2:**
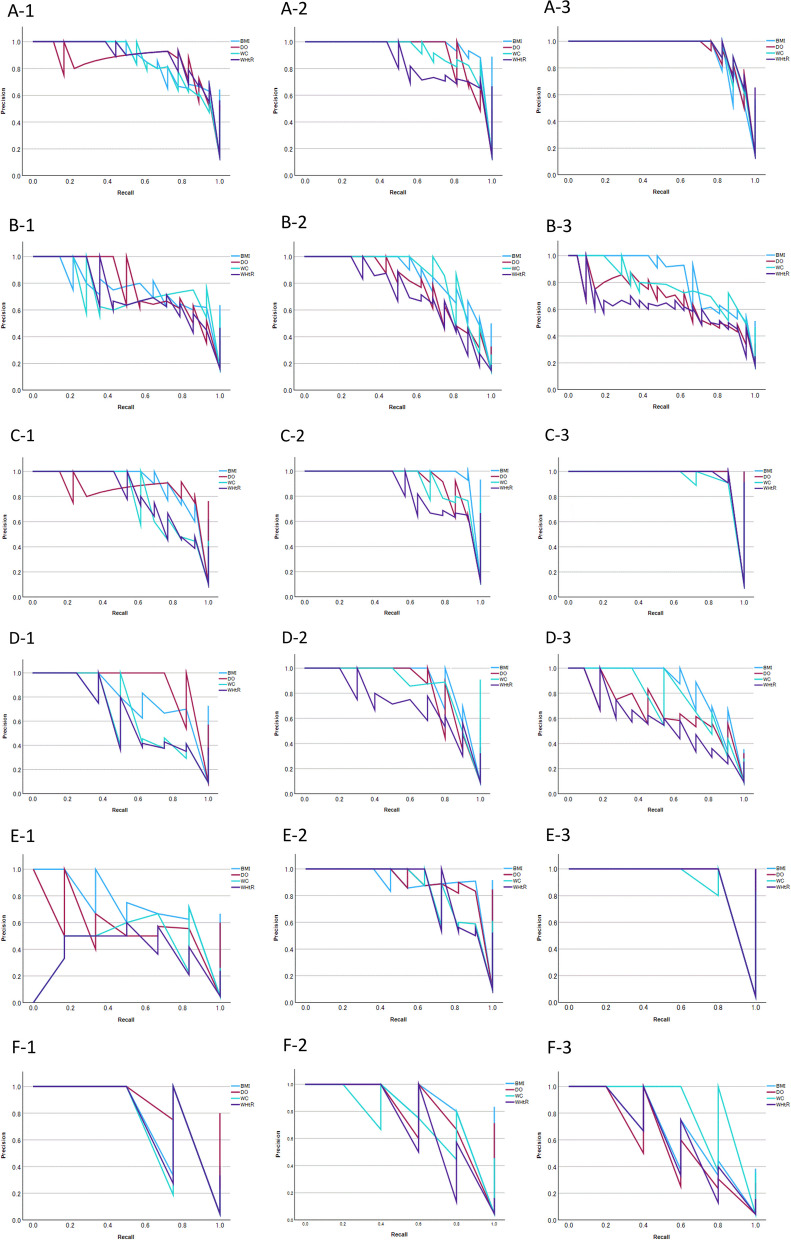
Precision-recall curves for body mass index (blue line), degree of obesity (red line), waist circumference (light blue line), and waist-to-height ratio (purple line) to identify obesity as determined by body fat percentage. A-1 to A-3 and B-1 to B-3, 85th percentile of body fat percentage; C-1 to C-3 and D-1 to D-3, 90th percentile of body fat percentage; and E-1 to E-3 and F-1 to F-3, 95th percentile of body fat percentage. Grade and sex are as follows: A-, C-, and E-1, 4th grade boys; A-, C-, and E-2, 5th grade boys; A-, C-, and E-3, 6th grade boys; B-, D-, and F-1, 4th grade girls; B-, D-, and F-2, 5th grade girls; and B-, D-, and F-3, 6th grade girls

Figures 1 and 2 display the ROC and PR curves, respectively, for BMI, degree of obesity, waist circumference, and waist-to-height ratio in distinguishing between ‘obesity’ and ‘normal’ status, as defined by body fat percentage. Tables 3 and 4 show the corresponding ROC and PR AUCs, respectively. The ROC AUCs and 95% CIs for BMI, degree of obesity, waist circumference, and waist-to-height ratio in identifying obesity—defined as body fat percentage > 85th, > 90th, and > 95th percentiles—were > 0.9 in nearly all cases for both sexes. The PR AUCs and 95% CIs for BMI and degree of obesity were > 0.8 in most instances. On the other hand, the PR AUCs and 95% CIs for waist circumference and waist-to-height ratio were < 0.8 in many cases.

**Table 3:** Area under the receiver operating characteristic curves for body mass index, degree of obesity, waist circumference, and waist-to-height ratio as indicators of whole-body percent fat in fourth to sixth grade Japanese school children

Whole body percent fat (%)	Measure	4th grade		5th grade		6th grade	
		boys (*n* = 124), girls (*n* = 87)		boys (*n* = 111), girls (*n* = 108)		boys (*n* = 114), girls (*n* = 116)	
		AUC	95% CI	AUC	95% CI	AUC	95% CI
85th percentile							
Boys	BMI	0.975	0.952—0.998	0.997	0.990—1.003	0.980	0.954—1.006
	DO	0.975	0.948—1.001	0.981	0.956—1.006	0.985	0.964—1.006
	WC	0.969	0.940—0.998	0.988	0.972—1.003	0.987	0.969—1.005
	WHtR	0.980	0.958—1.001	0.974	0.949—0.999	0.990	0.975—1.004
Girls	BMI	0.958	0.919—0.997	0.968	0.936—1.000	0.951	0.914—0.989
	DO	0.939	0.883—0.995	0.934	0.878—0.990	0.906	0.847—0.965
	WC	0.955	0.913—0.997	0.947	0.886—1.009	0.945	0.905—0.986
	WHtR	0.935	0.883—0.988	0.890	0.789—0.991	0.888	0.815—0.961
90th percentile							
Boys	BMI	0.989	0.974—1.004	0.999	0.997—1.002	1.000	1.000—1.000
	DO	0.990	0.974—1.005	0.987	0.970—1.005	1.000	1.000—1.000
	WC	0.963	0.929—0.998	0.990	0.976—1.004	0.997	0.991—1.004
	WHtR	0.963	0.927—0.998	0.976	0.952—1.001	0.999	0.996—1.002
Girls	BMI	0.983	0.958—1.007	0.989	0.971—1.007	0.974	0.939—1.009
	DO	0.991	0.970—1.011	0.971	0.933—1.010	0.953	0.909—0.997
	WC	0.937	0.875—0.999	0.995	0.985—1.005	0.954	0.905—1.003
	WHtR	0.938	0.882—0.995	0.959	0.914—1.004	0.919	0.853—0.984
95th percentile							
Boys	BMI	0.989	0.972—1.005	0.995	0.983—1.006	1.000	1.000—1.000
	DO	0.979	0.954—1.003	0.994	0.982—1.005	1.000	1.000—1.000
	WC	0.963	0.917—1.009	0.981	0.958—1.004	0.998	0.992—1.004
	WHtR	0.952	0.901—1.003	0.977	0.950—1.005	1.000	1.000—1.000
Girls	BMI	0.982	0.946—1.018	0.996	0.987—1.006	0.975	0.942—1.007
	DO	0.997	0.988—1.006	0.992	0.977—1.007	0.957	0.907—1.006
	WC	0.961	0.890—1.032	0.985	0.962—1.009	0.986	0.957—1.014
	WHtR	0.976	0.930—1.022	0.944	0.854—1.034	0.939	0.853—1.025

*AUC* area under curve,* BMI* body mass index, *CI* confidence interval, *DO *degree of obesity, *WC* waist circumference, *WHtR* waist-to-height ratio

**Table 4:** Area under the precision recall curves for body mass index, degree of obesity, waist circumference, and waist-to-height ratio as indicators of whole body percent fat in fourth to sixth grade Japanese school children

Whole body percent fat (%)	Measure	4th grade		5th grade		6th grade	
		boys (*n* = 124), girls (*n* = 87)		boys (*n* = 111), girls (*n* = 108)		boys (*n* = 114), girls (*n* = 116)	
		AUC	95% CI	AUC	95% CI	AUC	95% CI
85th percentile							
Boys	BMI	0.872	0.814–0.931	0.981	0.956–1.006	0.931	0.885–0.978
	DO	0.853	0.791–0.915	0.929	0.881–0.977	0.943	0.900–0.985
	WC	0.865	0.805–0.925	0.933	0.887–0.980	0.949	0.908–0.989
	WHtR	0.899	0.846–0.952	0.859	0.795–0.924	0.956	0.918–0.993
Girls	BMI	0.777	0.690–0.865	0.870	0.807–0.933	0.843	0.777–0.910
	DO	0.793	0.708–0.878	0.787	0.710–0.864	0.681	0.596–0.766
	WC	0.757	0.667–0.847	0.871	0.808–0.934	0.786	0.712–0.861
	WHtR	0.742	0.650–0.834	0.730	0.646–0.813	0.615	0.526–0.703
90th percentile							
Boys	BMI	0.926	0.879–0.972	0.995	0.982–1.008	1.000	1.000–1.000
	DO	0.881	0.823–0.938	0.936	0.890–0.981	1.000	1.000–1.000
	WC	0.813	0.745–0.882	0.936	0.890–0.981	0.974	0.944–1.003
	WHtR	0.809	0.740–0.878	0.864	0.800–0.928	0.992	0.976–1.008
Girls	BMI	0.829	0.750–0.908	0.925	0.876–0.975	0.869	0.808–0.930
	DO	0.944	0.896–0.993	0.870	0.806–0.933	0.682	0.597–0.766
	WC	0.696	0.599–0.792	0.947	0.905–0.990	0.775	0.699–0.851
	WHtR	0.666	0.567–0.765	0.742	0.659–0.825	0.585	0.495–0.674
95th percentile							
Boys	BMI	0.780	0.707–0.853	0.937	0.892–0.982	1.000	1.000–1.000
	DO	0.612	0.527–0.698	0.937	0.892–0.982	1.000	1.000–1.000
	WC	0.464	0.376–0.552	0.877	0.815–0.938	0.963	0.929–0.998
	WHtR	0.394	0.308–0.480	0.873	0.811–0.935	1.000	1.000–1.000
Girls	BMI	0.842	0.765–0.918	0.918	0.867–0.970	0.695	0.612–0.779
	DO	0.944	0.895–0.992	0.865	0.800–0.929	0.617	0.529–0.706
	WC	0.803	0.719–0.886	0.787	0.709–0.864	0.872	0.811–0.933
	WHtR	0.826	0.746–0.905	0.737	0.654–0.820	0.644	0.556–0.731

*AUC *area under curve, *BMI *body mass index, *CI* confidence interval, *DO* degree of obesity, *WC* waist circumference, *WHtR* waist-to-height ratio

Identification performance was evaluated using accuracy, precision, recall, F1 score (Table 5), as well as confusion matrices (Figs. S1–S3). The precision, recall, and F1 scores for BMI and degree of obesity in identifying obesity—defined as body fat percentage > 85th and > 90th percentiles—were > 70% in nearly all cases, while some F1 scores defined as body fat percentage > 95th percentile were < 70% in some cases.

**Table 5:** Accuracy, precision, recall, and F-1 scores between body mass index, degree of obesity, waist circumference, and waist-to-height ratio and current criteria in fourth to sixth grade Japanese school children

Whole body percent fat (%)	Measure	4th grade				5th grade				6th grade			
		boys (*n* = 124), girls (*n* = 87)				boys (*n* = 111), girls (*n* = 108)				boys (*n* = 114), girls (*n* = 116)			
		AC	PR	RC	F1	AC	PR	RC	F1	AC	PR	RC	F1
85th percentile													
Boys	BMI	0.855	0.500	1.000	0.667	0.973	0.933	0.875	0.903	0.965	0.882	0.882	0.882
	DO	0.952	0.875	0.778	0.824	0.910	1.000	0.375	0.545	0.956	0.929	0.765	0.839
	WC	0.927	0.846	0.611	0.710	0.946	0.917	0.688	0.786	0.965	0.933	0.824	0.875
	WHtR	0.855	0.500	1.000	0.667	0.919	0.706	0.750	0.727	0.956	0.833	0.882	0.857
Girls	BMI	0.920	0.769	0.714	0.741	0.917	0.769	0.625	0.690	0.897	0.737	0.667	0.700
	DO	0.897	1.000	0.357	0.526	0.907	1.000	0.375	0.545	0.871	0.875	0.333	0.483
	WC	0.874	1.000	0.214	0.353	0.917	1.000	0.438	0.609	0.862	0.778	0.333	0.467
	WHtR	0.885	0.667	0.571	0.615	0.898	0.727	0.500	0.593	0.853	0.700	0.333	0.452
90th percentile													
Boys	BMI	0.815	0.361	1.000	0.531	0.973	0.867	0.929	0.897	0.947	0.647	1.000	0.786
	DO	0.960	0.750	0.923	0.828	0.928	1.000	0.429	0.600	0.974	0.786	1.000	0.880
	WC	0.919	0.615	0.615	0.615	0.946	0.833	0.714	0.769	0.965	0.733	1.000	0.846
	WHtR	0.815	0.361	1.000	0.531	0.919	0.647	0.786	0.710	0.939	0.611	1.000	0.759
Girls	BMI	0.943	0.615	1.000	0.762	0.935	0.615	0.800	0.696	0.914	0.526	0.909	0.667
	DO	0.966	1.000	0.625	0.769	0.963	1.000	0.600	0.750	0.922	0.625	0.455	0.526
	WC	0.943	1.000	0.375	0.545	0.954	0.857	0.600	0.706	0.931	0.667	0.545	0.600
	WHtR	0.885	0.417	0.625	0.500	0.935	0.636	0.700	0.667	0.922	0.600	0.545	0.571
95th percentile													
Boys	BMI	0.758	0.167	1.000	0.286	0.964	0.733	1.000	0.846	0.895	0.294	1.000	0.455
	DO	0.919	0.375	1.000	0.545	0.955	1.000	0.545	0.706	0.921	0.357	1.000	0.526
	WC	0.927	0.385	0.833	0.526	0.937	0.667	0.727	0.696	0.912	0.333	1.000	0.500
	WHtR	0.758	0.167	1.000	0.286	0.910	0.529	0.818	0.643	0.886	0.278	1.000	0.435
Girls	BMI	0.897	0.308	1.000	0.471	0.926	0.385	1.000	0.556	0.879	0.263	1.000	0.417
	DO	0.989	0.800	1.000	0.889	0.972	0.667	0.800	0.727	0.940	0.375	0.600	0.462
	WC	0.989	1.000	0.750	0.857	0.963	0.571	0.800	0.667	0.948	0.444	0.800	0.571
	WHtR	0.908	0.333	1.000	0.500	0.926	0.364	0.800	0.500	0.940	0.400	0.800	0.533

*AC* accuracy, *PR* precision, *RC* recall, *F1* F1 score, *BMI* body mass index, *CI* confidence interval, *DO *degree of obesity, *WC* waist circumference, *WHtR* waist-to-height ratio

Cohen’s kappa coefficients and MCCs are shown in Table 6. Regarding Cohen’s kappa, substantial (0.61–0.80) and almost perfect (0.81–1.00) agreements were observed between BMI and body fat percentage defined at the 85th or 90th percentile, while moderate agreement was more often observed for the degree of obesity. In contrast, fair agreement (0.21–0.40) was observed between BMI or the degree of obesity and body fat percentage defined at the 95th percentile. MCC values were generally consistent with the kappa coefficients.

**Table 6:** Cohen's kappa coefficients and Matthews correlation coefficients between body mass index, degree of obesity, waist circumference, and waist-to-height ratio and current criteria in fourth to sixth grade Japanese school children

Whole body percent fat (%)	Measure	4th grade		5th grade		6th grade	
		boys (*n* = 124),	girls (*n* = 87)	boys (*n* = 111),	girls (*n* = 108)	boys (*n* = 114),	girls (*n* = 116)
		Kappa	MCC	Kappa	MCC	Kappa	MCC
85th percentile							
Boys	BMI	0.587	0.644	0.888	0.888	0.862	0.862
	DO	0.796	0.797	0.507	0.582	0.814	0.819
	WC	0.669	0.681	0.756	0.766	0.855	0.857
	WHtR	0.587	0.644	0.680	0.680	0.831	0.832
Girls	BMI	0.693	0.694	0.642	0.647	0.638	0.639
	DO	0.482	0.564	0.505	0.582	0.425	0.491
	WC	0.314	0.432	0.570	0.631	0.402	0.449
	WHtR	0.548	0.551	0.537	0.549	0.379	0.414
90th percentile							
Boys	BMI	0.445	0.535	0.881	0.882	0.757	0.781
	DO	0.805	0.811	0.567	0.629	0.865	0.873
	WC	0.570	0.570	0.739	0.742	0.827	0.840
	WHtR	0.445	0.535	0.663	0.667	0.726	0.755
Girls	BMI	0.731	0.759	0.660	0.667	0.621	0.652
	DO	0.752	0.776	0.731	0.759	0.485	0.493
	WC	0.521	0.594	0.682	0.694	0.563	0.566
	WHtR	0.438	0.449	0.631	0.632	0.529	0.530
95th percentile							
Boys	BMI	0.221	0.353	0.826	0.839	0.415	0.512
	DO	0.511	0.586	0.684	0.721	0.494	0.572
	WC	0.493	0.536	0.661	0.661	0.465	0.550
	WHtR	0.221	0.353	0.594	0.612	0.393	0.495
Girls	BMI	0.431	0.524	0.524	0.596	0.374	0.480
	DO	0.883	0.889	0.713	0.716	0.431	0.445
	WC	0.851	0.861	0.648	0.658	0.546	0.573
	WHtR	0.463	0.549	0.466	0.509	0.505	0.540

*BMI* body mass index, *DO* degree of obesity, *WC* waist circumference, *WHtR* waist-to-height ratio, *MCC *Matthews correlation coefficient

## Discussion

This study investigated how well BMI, degree of obesity, waist circumference, and waist-to-height ratio—easily measured in school settings—relate to obesity as defined by body fat. Among these four indices, BMI follows established thresholds: values ≥ 25 and ≥ 30 indicate overweight and obesity, respectively, in adults, regardless of age or sex. In children, sex- and age-specific BMI values equivalent to adult BMI thresholds are used as reference standards [9]. In contrast, the degree of obesity is defined as an actual weight increase of ≥ 20% over the standard weight, irrespective of age or sex, although the standard weight itself is calculated based on sex and age [31]. Therefore, this study assessed the associations between these four indices and body fat percentage, stratified by sex and age.

The analysis of correlations between the four indices (i.e., BMI, degree of obesity, waist circumference, and waist-to-height ratio) and body fat mass, fat-free mass, and body fat percentage revealed that BMI, degree of obesity, and waist circumference were significantly positively associated with body fat mass and percentage in both sexes. Waist-to-height ratio showed a significant positive correlation with body fat mass and percentage only in boys. These findings confirmed linear associations between all four indices and body fat mass and percentage, though the strength of association varied by sex.

In the present study, ROC and PR curves were constructed to evaluate the performance of BMI, degree of obesity, waist circumference, and waist-to-height ratio in identifying obesity defined by body fat percentage. PR AUCs were lower for waist circumference and waist-to-height ratio. BMI and degree of obesity also showed decreased AUCs at the 95th percentile threshold for body fat percentage, though the decline was less pronounced. It has been noted that in populations with few positives and a severe class imbalance (positive and negative), both the model and the measures used to evaluate its performance may be biased toward the majority class [38–40]. PR curve analysis is known to be robust for unbalanced data sets [41–43] and provides more informative results for classifying distributions containing unbalanced data than ROC curve analysis [44]. This was relevant in the present study, where true positive rates were 5%, 10%, and 15%, respectively. The findings suggest that BMI and degree of obesity are reliable indicators of body fat percentage, irrespective of threshold.

The present study evaluated the concordance between different indicators of obesity (i.e., BMI, degree of obesity, waist circumference, and waist-to-height ratio) and body fat percentage using various statistical measures, including confusion matrix, accuracy, precision, recall, F1 score, Cohen’s kappa coefficient, and MCC. Accuracy exceeded 0.9 in most cases. For BMI, precision, recall, and F1 scores were ≥ 0.7 when obesity was defined as body fat percentage exceeding the 85th or 90th percentile. Degree of obesity showed similarly high performance at the 90th percentile. Kappa agreement and MCC supported these results. In contrast, waist circumference and waist-to-height ratio had lower values. These results suggest that BMI and degree of obesity are more accurate in identifying obesity at the 85th and 90th percentile thresholds.

In the present study, BMI and degree of obesity showed favorable ROC and PR curve fits in each sex and grade. Body fat levels in growing children vary by age and differ between girls and boys [45, 46]. Therefore, it is essential to consider growth when defining obesity. This also applies when using BMI in pediatric populations. The present study used obesity criteria proposed by Cole [9], which may have influenced the sex- and grade-stratified ROC curve results. On the other hand, obesity is defined as a degree of obesity ≥ 20%, independent of age or sex [31]. On the other hand, because the standard body weight, which is the basis for calculating the degree of obesity, is calculated according to sex, age, and height [31], sex and age are also taken into account when determining obesity. Waist circumference and waist-to-height ratio were judged using fixed thresholds of 75 cm and 0.5, respectively, without regard for age or sex. This may explain why BMI and degree of obesity showed stronger agreement with body fat percentage when analyzed by grade and sex. In addition, both BMI and degree of obesity are derived from height and weight, albeit through different formulas. Because this study used total body fat percentage, this may have also contributed to the favorable ROC and PR curve fits and concordance in classification.

As obesity involves excess body fat accumulation, body fat percentage should be a central criterion. While DXA and underwater body weight measurements are used to determine body fat, facilities and measurement capabilities are limited [17]. Although single-frequency bioelectrical impedance analyzers are currently widely available and inexpensive, concerns remain regarding their accuracy [18–21]. Multi-frequency analyzers, although more precise, are impractical for large-scale student assessments. Hence, these methods are typically restricted to research, and sufficient data to establish diagnostic criteria remain lacking. Accordingly, the present study used percentiles of body fat percentage for each sex and grade to determine obesity conveniently. While ROC curve analysis provides cut-off values and fit metrics via AUC, such thresholds lack clinical significance in the absence of validated diagnostic criteria for body fat percentage. The results of the present study showed that BMI and degree of obesity performed consistently well at the 85th, 90th, and 95th percentile thresholds. These findings indicate that BMI and degree of obesity can still be used once body fat–based obesity criteria are formalized.

However, under current criteria, BMI and degree of obesity performed well at the 90th percentile but less so at the 95th percentile or when alternative indices were used. Therefore, if simple indicators like BMI and degree of obesity are to be used consistently, it will be necessary to review obesity classification thresholds based on these indices when formal criteria are established.

## Limitations

First, while this study was conducted in a population-based sample, the sample size was relatively small. This limitation may have influenced the cutoff values identified for determining obesity based on BMI, waist circumference, and waist-to-height ratio. Some results were not particularly favorable for determining obesity based on body fat, underscoring the importance of examining cutoff values in larger populations.

Second, not all participants were measured at the same time of day, which may have introduced diurnal variations in the results. However, Andersen et al. assessed diurnal variation using a multi-frequency BIA device (Xitron 4200), which differs from the device used in the present study, and found variation in the range of 1.1–2.8% [47].

Third, factors such as hydration status, presence or absence of menarche, menstrual cycle phase, room temperature, and humidity were not measured. Since the BIA method can be influenced by these factors, they may have impacted the results of this study.

## Conclusions

The present results revealed that BMI and degree of obesity performed well across all three body fat percentage cases (85th, 90th, and 95th percentiles). However, the current BMI and degree of obesity criteria for determining obesity were effective at the 90th percentile of body fat percentage, but less so for the other indicators and at the 95th percentile. These findings suggest that BMI and degree of obesity criteria are similar to those based on body fat percentage. Nevertheless, further investigation is needed to refine the criteria for determining obesity using BMI, degree of obesity, or body fat percentage as measured by BIA.

## Supplementary Information

Supplementary Material 1: Figure S1. Confusion matrix between predictors and obesity as determined by body fat percentage exceeding the 85th percentile of body fat percentage. Figure S2. Confusion matrix between predictors and obesity as determined by body fat percentage exceeding the 90th percentile of body fat percentage. Figure S3. Confusion matrix between predictors and obesity as determined by body fat percentage exceeding the 95th percentile of body fat percentage.

## Data Availability

The dataset generated during the current study is available from the corresponding author upon reasonable request.

## Abbreviations

AUC: Area under the curve
BIA: Bioelectrical impedance analysis
BMI: Body mass index
CI: Confidence interval
DXA: Dual-energy X-ray absorptiometry
MCC: Matthews correlation coefficient
PR: Precision-recall
ROC: Receiver operating characteristic

## Declarations

### Competing for publication

No applicable.
