# Supplementary material for: Association between anthropometric indices and body fat for identifying excess body fat in elementary school children: a population-based cross-sectional study
Source: J Physiol Anthropol. 2025 Nov 19;44:31. doi: 10.1186/s40101-025-00410-w (PMC12628560; doi:10.1186/s40101-025-00410-w)
Supplement: Supplementary file 1 — Supplementary Material 1: Figure S1. Confusion matrix between predictors and obesity as determined by body fat percentage exceeding the 85th percentile of body fat percentage. Figure S2. Confusion matrix between predictors and obesity as determined by body fat percentage exceeding the 90th percentile of body fat percentage. Figure S3. Confusion matrix between predictors and obesity as determined by body fat percentage exceeding the 95th percentile of body fat percentage. [file 40101_2025_410_MOESM1_ESM.docx]

**Supplementary Online Content**

Ohara K, et al., Association between anthropometric indices and body fat for identifying excess body fat in elementary school children: a population-based cross-sectional study.

**Fig S1. Confusion matrix between predictors and obesity as determined by body fat percentage exceeding the 85th percentile of body fat percentage.**

**Fig S2. Confusion matrix between predictors and obesity as determined by body fat percentage exceeding the 90th percentile of body fat percentage.**

**Fig S3. Confusion matrix between predictors and obesity as determined by body fat percentage exceeding the 95th percentile of body fat percentage.**


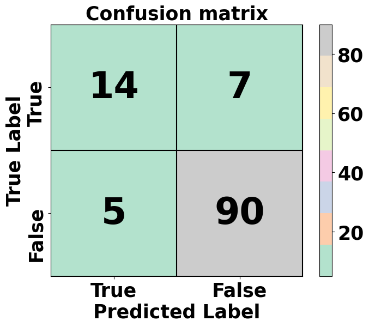

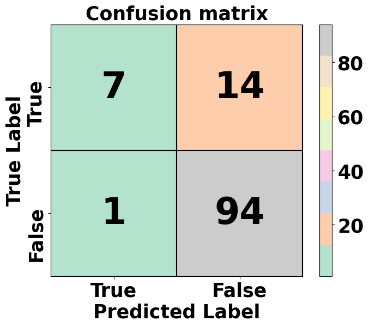

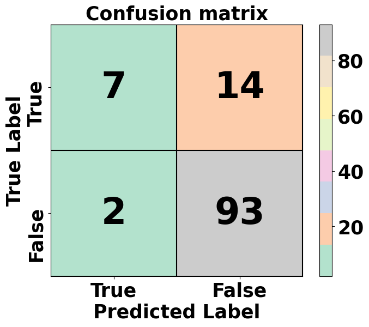

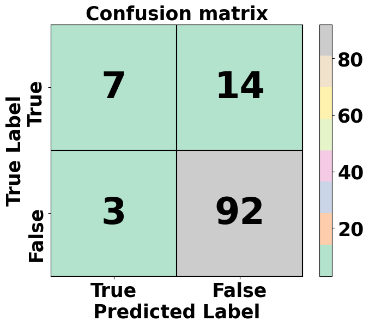

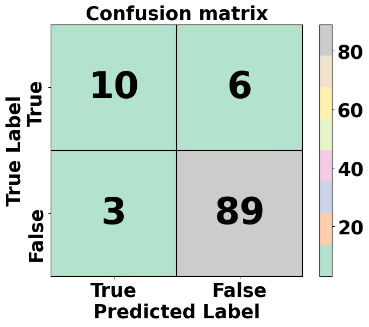

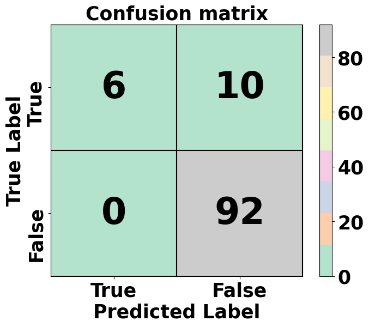

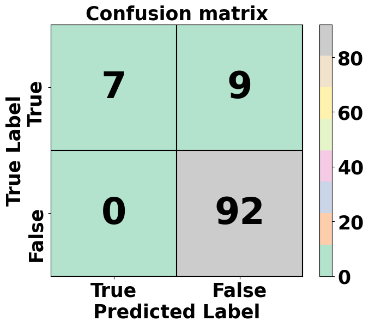

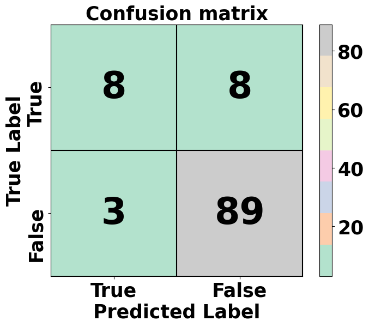

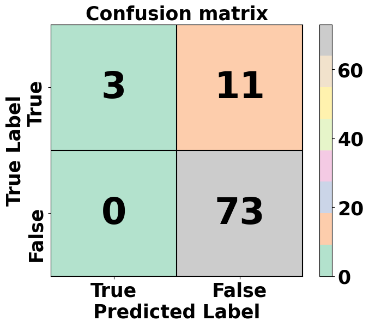

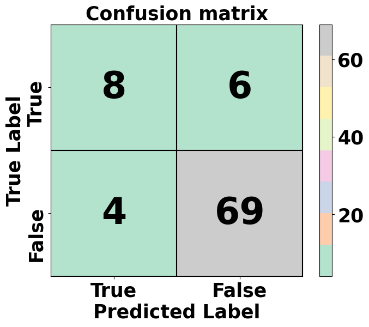

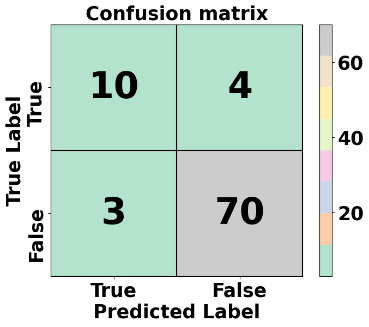

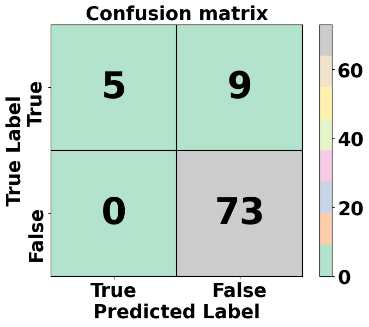

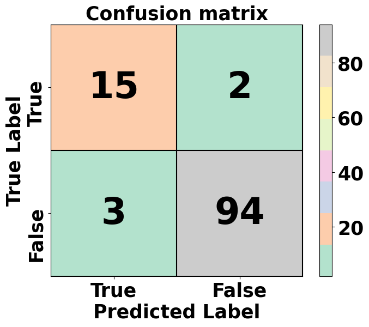

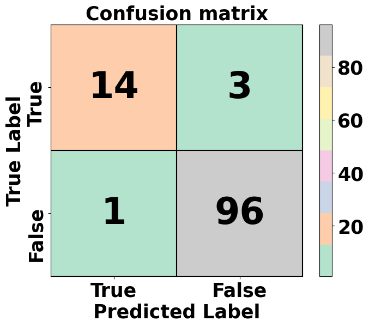

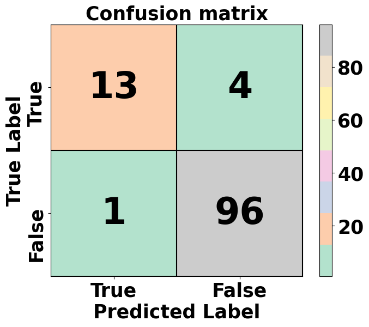

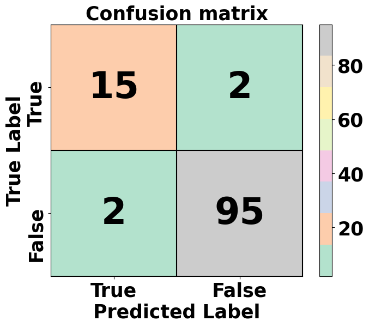

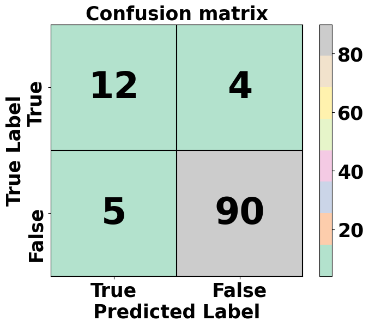

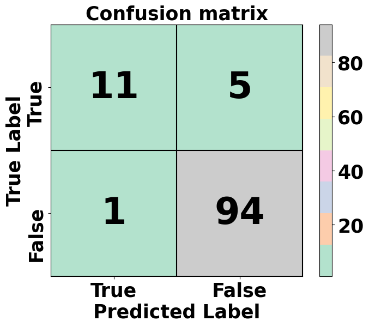

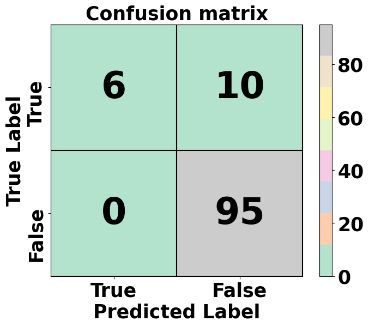

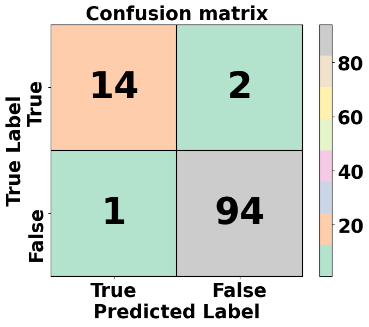

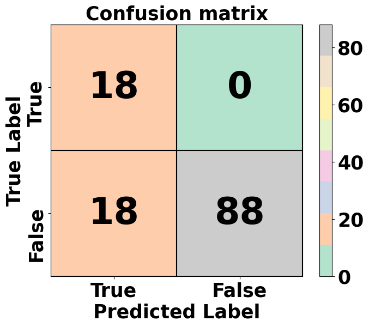

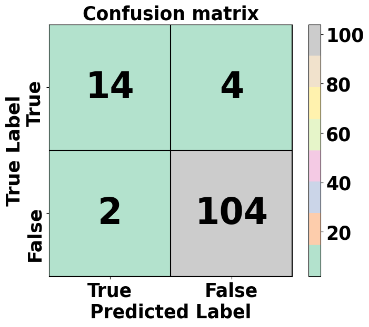

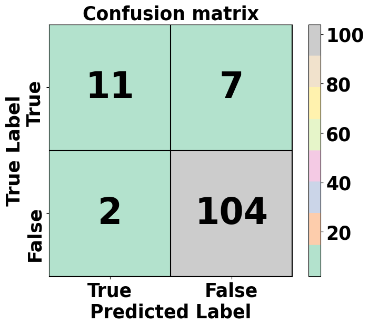

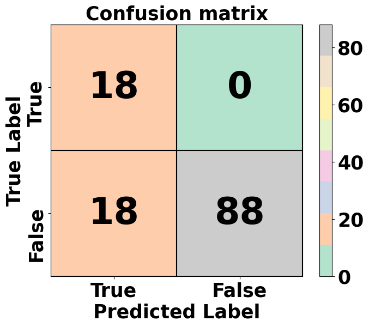


D-3

D-4

D-5

D-6

D-2

D-1

C-3

C-4

C-5

C-6

C-2

C-1

B-1

B-2

B-6

B-5

B-4

B-3

A-1

A-6

A-5

A-4

A-3

A-2

**Fig S1. Confusion matrix between predictors and obesity as determined by body fat percentage exceeding the 85th percentile of body fat percentage. Predictors of A-1 to A-6, B-1 to B-6, C-1 to C-6, and D-1 to D-6 are BMI, degree of obesity, waist circumference, and waist-to-height ratio, respectively. Grade and sex are as follows: A-, B-, C-, and D-1, 4th grade boys; A-, B-, C-, and D-2, 5th grade boys; A-, B-, C-, and D-3, 6th grade boys; A-, B-, C-, and D-4, 4th grade girls; A-, B-, C-, and D-5, 5th grade girls; and A-, B-, C-, and D-6, 6th grade girls.**

D-3

D-4

D-5

D-6

D-2

D-1

C-3

C-4

C-5

C-6

C-2

C-1

B-1

B-2

B-6

B-5

B-4

B-3

A-1

A-6

A-5

A-4

A-3

A-2


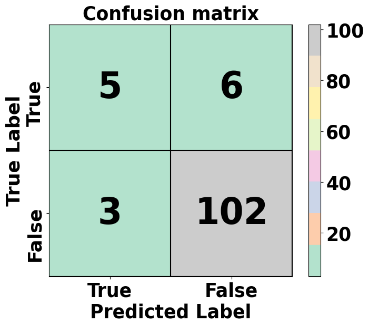

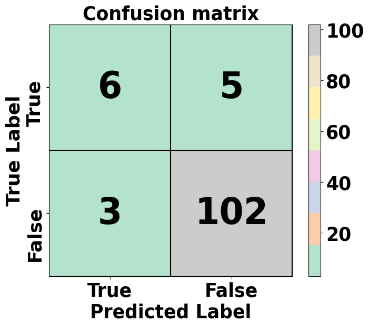

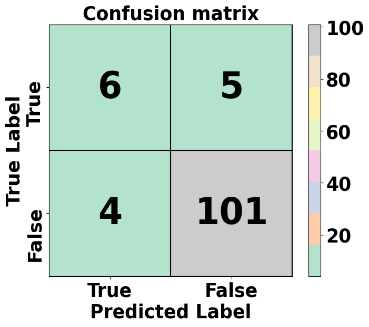

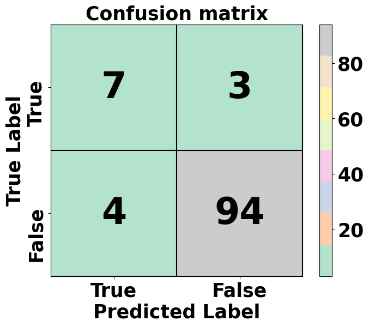

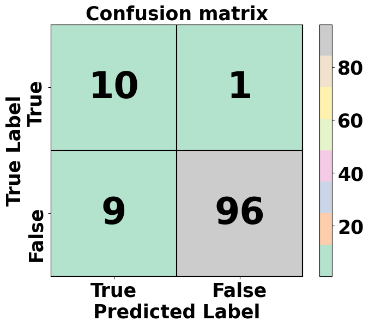

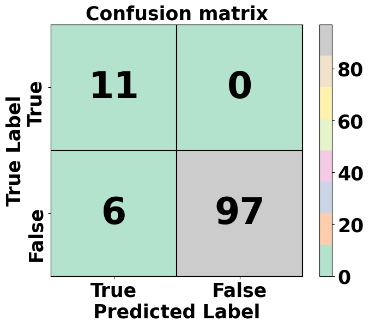

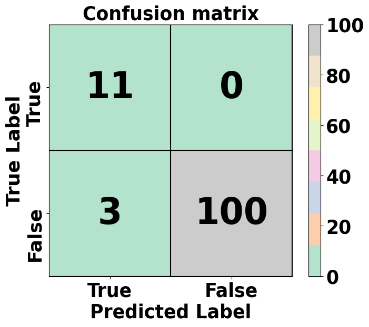

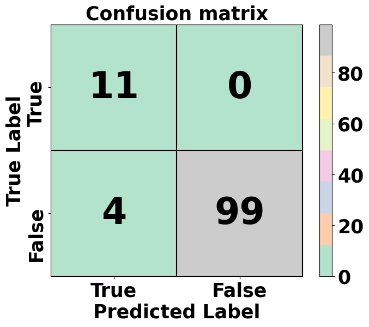

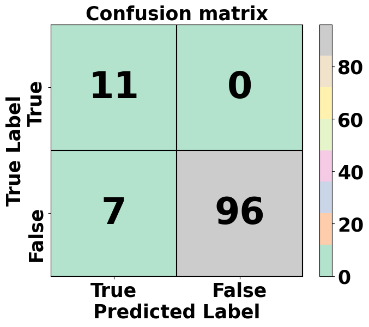

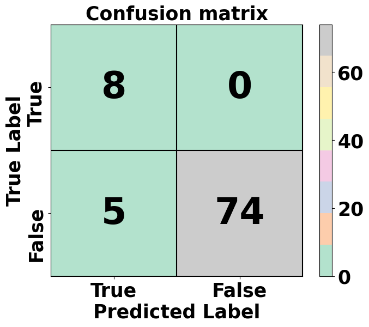

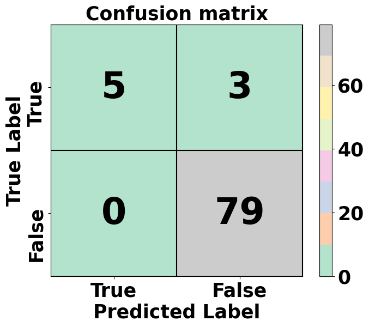

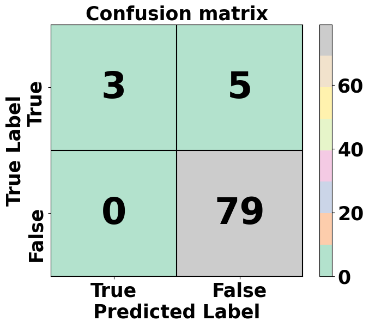

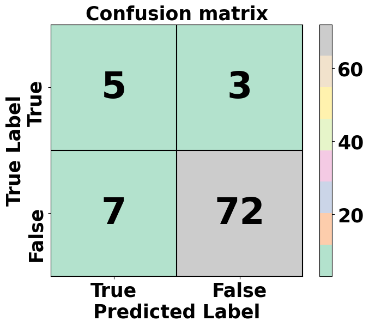

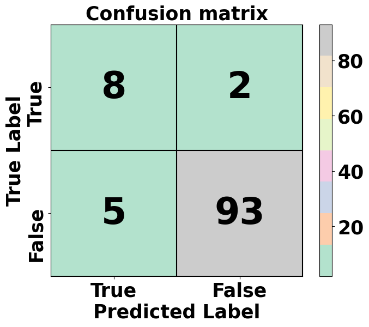

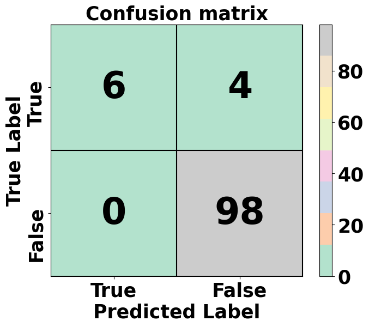

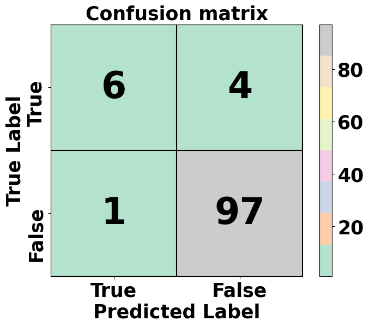

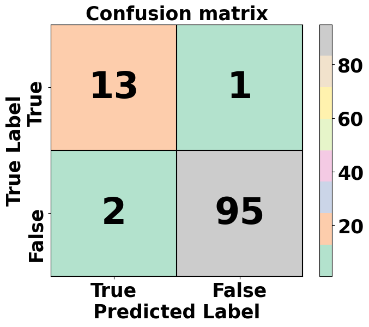

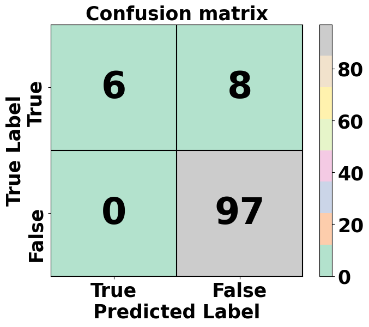

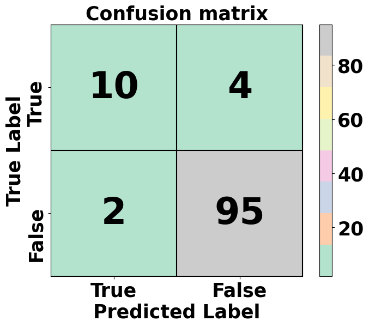

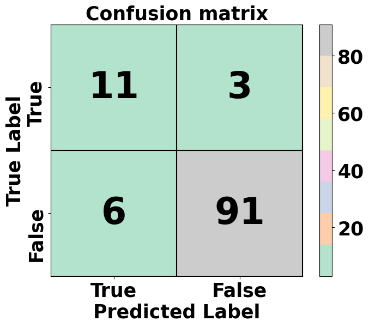

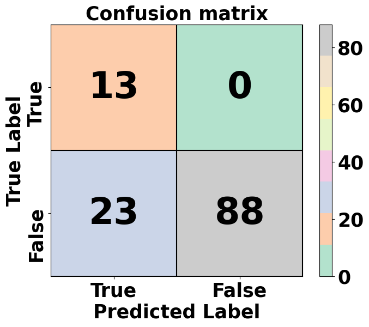

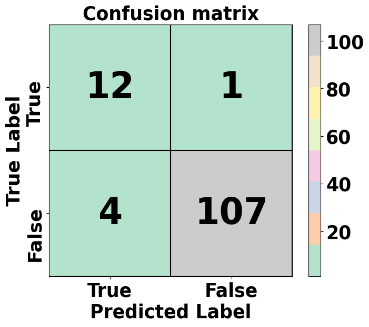

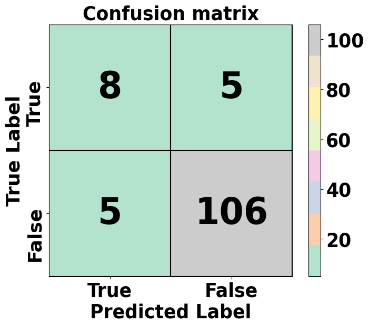

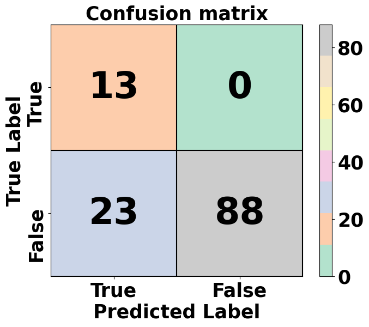


**Fig S2. Confusion matrix between predictors and obesity as determined by body fat percentage exceeding the 90th percentile of body fat percentage. Predictors of A-1 to A-6, B-1 to B-6, C-1 to C-6, and D-1 to D-6 are BMI, degree of obesity, waist circumference, and waist-to-height ratio, respectively. Grade and sex are as follows: A-, B-, C-, and D-1, 4th grade boys; A-, B-, C-, and D-2, 5th grade boys; A-, B-, C-, and D-3, 6th grade boys; A-, B-, C-, and D-4, 4th grade girls; A-, B-, C-, and D-5, 5th grade girls; and A-, B-, C-, and D-6, 6th grade girls.**

D-3

D-4

D-5

D-6

D-2

D-1

C-3

C-4

C-5

C-6

C-2

C-1

B-1

B-2

B-6

B-5

B-4

B-3

A-1

A-6

A-5

A-4

A-3

A-2


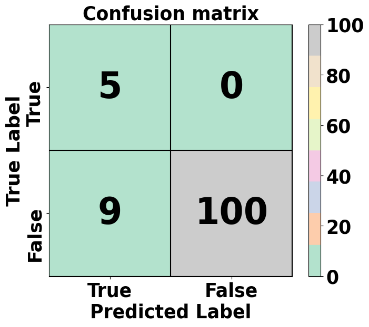

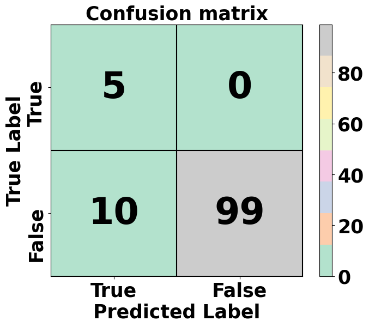

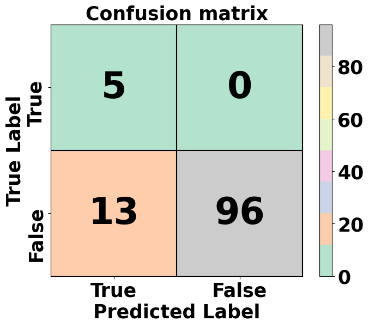

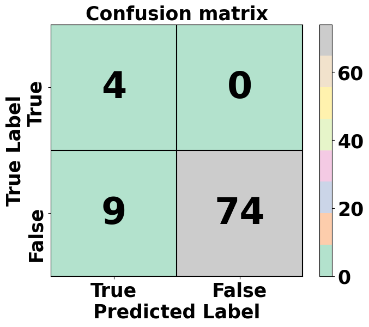

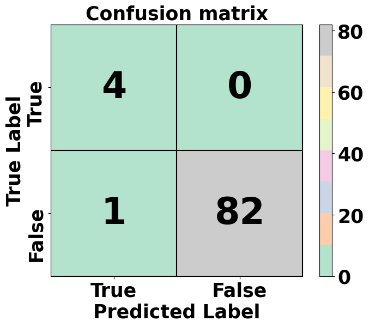

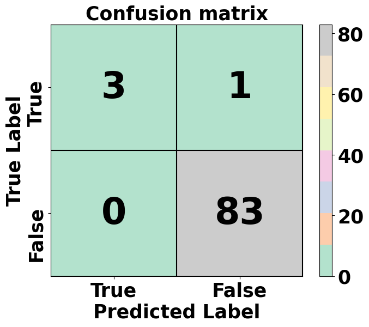

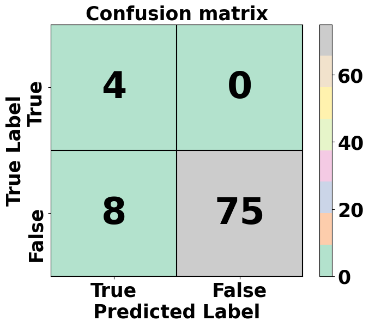

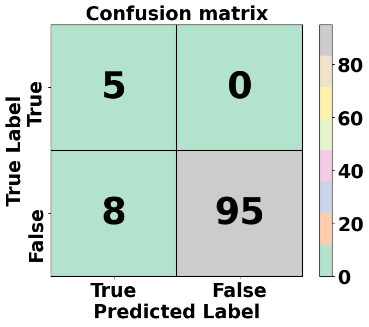

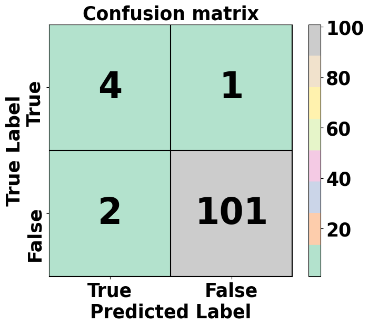

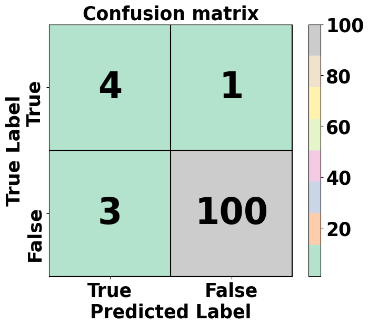

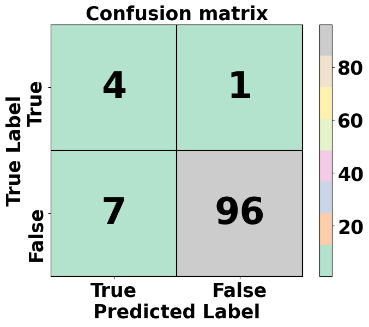

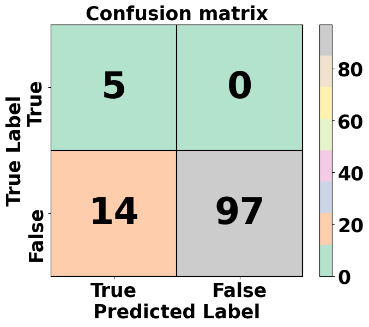

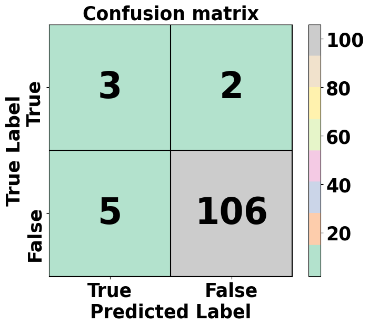

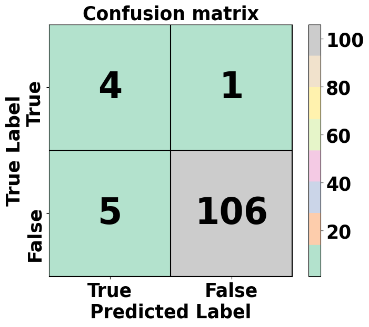

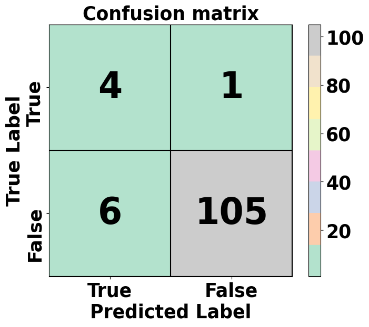

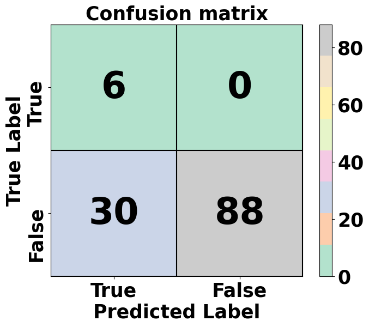

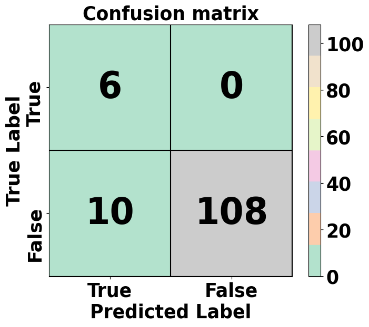

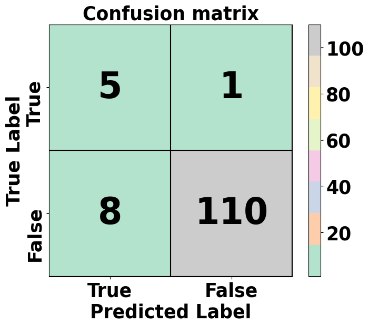

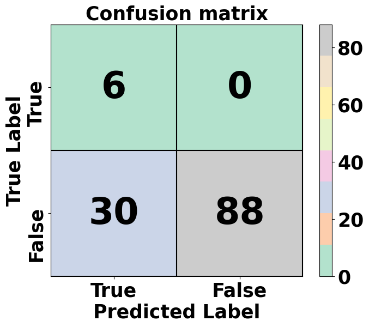

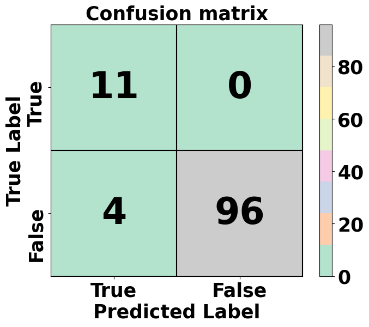

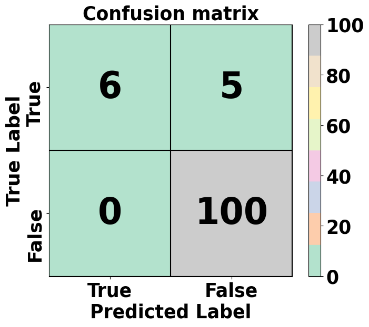

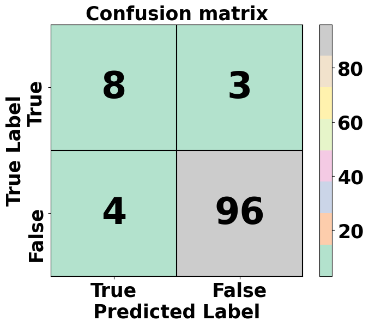

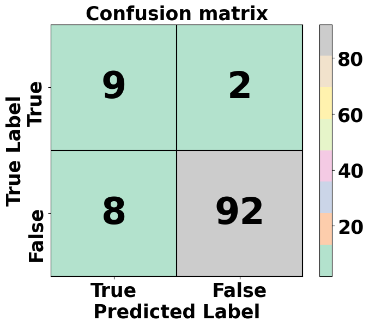

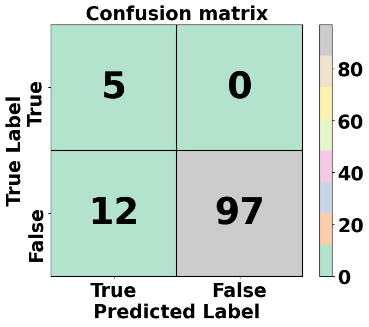


**Fig S3. Confusion matrix between predictors and obesity as determined by body fat percentage exceeding the 95th percentile of body fat percentage. Predictors of A-1 to A-6, B-1 to B-6, C-1 to C-6, and D-1 to D-6 are BMI, degree of obesity, waist circumference, and waist-to-height ratio, respectively. Grade and sex are as follows: A-, B-, C-, and D-1, 4th grade boys; A-, B-, C-, and D-2, 5th grade boys; A-, B-, C-, and D-3, 6th grade boys; A-, B-, C-, and D-4, 4th grade girls; A-, B-, C-, and D-5, 5th grade girls; and A-, B-, C-, and D-6, 6th grade girls.**
